# Selective outcome reporting and non-reporting in trials of psychedelic drugs for mental disorders

**DOI:** 10.1192/j.eurpsy.2025.393

**Published:** 2025-08-26

**Authors:** I. A. Cristea, G. Tomei, J. Freddi, A. Bonessio

**Affiliations:** 1 General Psychology, University of Padova, Padova, Italy

## Abstract

**Introduction:**

Selective reporting of outcome data (SOR) refers to trialists selecting results for publication based on the results of a subset of the initially measured outcomes. SOR for specific, potentially very profitable, treatments, particularly in key junctures such as in the period leading to or immediately preceding approval, has not been examined.

**Objectives:**

We examined the prevalence and types of SOR for three of the most prominent psychedelic drugs, either approved by regulators or considered very close to approval: esketamine, psilocybin and MDMA.

**Methods:**

We used a publicly available inception cohort (https://osf.io/yfv9n) of intervention trials of psychedelic drugs that were registered on clinicaltrials.gov by March 2023. We selected randomized trials in participants with symptoms, a diagnosis or risk of mental disorders. Trials had to assess the efficacy of esketamine, psilocybin or MDMA, alone or in combination with other treatments, compared to any control or active intervention, and include at least one efficacy outcome.

**Results:**

We identified 98 randomized trials, 56 of which had a clinicaltrials.gov status of completed, terminated or unknown as of July 2024. Sixteen of 56 (28.5%) had no publication available as well as no results posted on clinicaltrials.gov. Of these sixteen, seven were described as completed in the registry (three had a completion date in 2022, two in 2023). Another 8 trials we described as unknown, with anticipated completion dates ranging from 2021 to 2023. For 29 trials (51%) we could identify peer-reviewed publications. Five other trials had only been published as conference posters or company press-releases. Of the 29 trials matched with publications, the primary outcome measure had been changed in 2 (7%), with an outcome initially registered as secondary upgraded to primary. There were changes regarding the timepoint of assessment for the primary outcome in 7 trials (24%): in 4 trials the timepoint had been changed, while in 3 trials, the publication only reported on a subset of the timepoints registered for the primary outcome.

**Conclusions:**

Selective reporting and non-reporting of study results are present in trials of the most prominent psychedelic drugs, but given the scarce information contained in clinical trial registries, they are difficult to assess. Full access to all time-stamped versions of trial protocols and statistical analysis plans would be necessary to gauge the extent and types of SOR, including changes to the analysis method.

**Disclosure of Interest:**

None Declared

